# Design suggestions for a persuasive e-coaching application: A study on informal caregivers’ needs

**DOI:** 10.1177/20552076231177129

**Published:** 2023-05-30

**Authors:** Shweta Premanandan, Awais Ahmad, Åsa Cajander, Pär Ågerfalk, Lisette van Gemert-Pijnen

**Affiliations:** 1 Department of Informatics and Media, 8097 Uppsala University, Uppsala, Sweden; 2 Department of Information Technology, 8097 Uppsala University, Uppsala, Sweden; 3 Department of Psychology, Health, and Technology, Faculty of Behavioral, Management and Social Sciences, 3230 University of Twente, Enschede, The Netherlands

**Keywords:** E-coaching, informal caregivers, e-health, designing application, user needs, persuasive system design, online intervention

## Abstract

**Objective:**

Informal caregivers such as relatives or close friends of patients are essential for caregiving at home. However, caregiving is a complex experience that may affect the caregivers’ well-being. Therefore, there is a need to provide support for caregivers, which we address in this article by proposing design suggestions for an e-coaching application. This study identifies the unmet needs of caregivers in Sweden and provides design suggestions for an e-coaching application using the persuasive system design (PSD) model. The PSD model offers a systematic approach to designing IT interventions.

**Methods:**

A qualitative research design was used, and semi-structured interviews were conducted with 13 informal caregivers from different municipalities in Sweden. A thematic analysis was performed to analyze the data. The PSD model was used to map the needs emerging from this analysis to propose design suggestions for an e-coaching application for caregivers.

**Results:**

Six needs were identified, and based on them, we proposed design suggestions for an e-coaching application using the PSD model. These unmet needs are monitoring and guidance, assistance to avail formal care services, access to practical information without being overwhelmed, feeling of community, access to informal support, and grief acceptance. The last two needs could not be mapped using the existing PSD model, resulting in an extended PSD model.

**Conclusion:**

This study revealed the important needs of informal caregivers based on which design suggestions for an e-coaching application were presented. We also proposed an adapted PSD model. This adapted PSD model can be further used for designing digital interventions in caregiving.

## Introduction

Informal caregivers are important to the effective functioning of traditional healthcare in our society.^1,2^ Often, they have taken over much of the burden of providing care at home, risking reduced well-being. Informal caregivers (hereafter referred to as caregivers) are friends or relatives who provide care for a person with a long-term chronic illness or disability that goes beyond the care needs of their usual relationship.^3^ In Europe alone, around 80% of long-term care is provided by caregivers.^4^ This number is expected to increase due to an increasingly older population and policies favoring deinstitutionalization and outpatient care.^5,6^

Against this backdrop, there has been a shift toward home-based, informal care, whereby the formal healthcare system is beginning to rely more on the support from caregivers to provide care to their relatives or friends. Caregivers’ experience may vary based on multiple factors such as motivation to provide care, the intensity of caregiving tasks, frequency, skills, care situation, and any unmet needs of caregivers.^7^ Additionally, caregivers may experience social isolation and financial difficulties.^8^ Due to these physical, psychosocial, and financial strains, caregivers can experience an increased burden.^9^ Caregiving also provides caregivers with positive emotions, such as finding meaning in the caregiving process and feelings of personal growth.^10^ In short, caregiving can be quite a complex experience that may affect the well-being of caregivers.^11^ Consequently, there is an urgent need to provide caregivers with assistance and support to continue providing care.

E-coaching is a promising e-health research direction to address the needs of caregivers.^12^ An e-coaching application is a digital entity engaged in a continuous dialogue with the user to set goals and promote goal striving through persuasive techniques.^13^ It can also observe, learn, and predict user behavior over time to aid in coaching.^14^ E-coaching applications are adaptive in nature and context-aware and are engaging while using persuasive techniques. In this context of caregivers, it is to support caregivers to continue to provide care and empower them in this process. Caregivers find such online interventions suitable as such support can transcend geographical barriers.^15^ For this study, e-coaching application is an information technology (IT)-based programmable application designed to support caregivers in their caregiving activities and engage in self-care. Healthcare professionals are not directly involved in coaching caregivers via this application.

In this study, we provide design suggestions for such an e-coaching application using the persuasive system design (PSD) model.^16^ Persuasive designing is the process of designing IT applications with the aim of influencing the users’ behavior or attitudes with their consent and by being transparent with the intent of persuasion.^16,17^ When designing IT applications for informal caregivers, persuasive designing is relevant as it can help encourage and motivate caregivers to foster actions and behaviors that may improve their caregiving experience and well-being. Persuasive designing can help encourage caregivers to adopt the IT application by making it appealing, easy to use, and demonstrating the benefits of using it.^16^ It can also help increase engagement by providing feedback and praise that reinforce positive behaviors. For instance, the application could provide reminders and alerts to help caregivers stay updated on their caregiving tasks and provide positive feedback when tasks are completed. Persuasive designing can also help improve caregiver well-being by promoting self-care.^18^ It can support behavior change by providing resources on best practices for caregiving and offering personalized recommendations based on the caregiver's needs and preferences.^19,20^ This personalized approach can help caregivers feel more supported and motivated to prioritize their own well-being.^21^

The PSD model is a comprehensive framework developed to aid in the design of systems capable of influencing users’ behavior.^22^ It is more relevant than other persuasive design approaches as it combines PSD principles and behavior change techniques into design features that support end-user values and needs for a long-term behavior change.^23,24^ It is provides designers a systematic approach to designing persuasive IT applications that are tailored to the specific needs of caregivers, making them more effective in helping them achieve their goals. It also provides a systematic approach to designing engaging and usable interventions.^25^ Additionally, the PSD model provides structured design principles that can be used by designers compared with other models such as the Fogg behavioral model and the Hook model.^26,27^ It also addresses the ethics of persuasive designing by providing seven persuasive designing postulates that outline autonomy and transparency as important aspects to consider when designing a persuasive system.^16^

**Figure 1.**
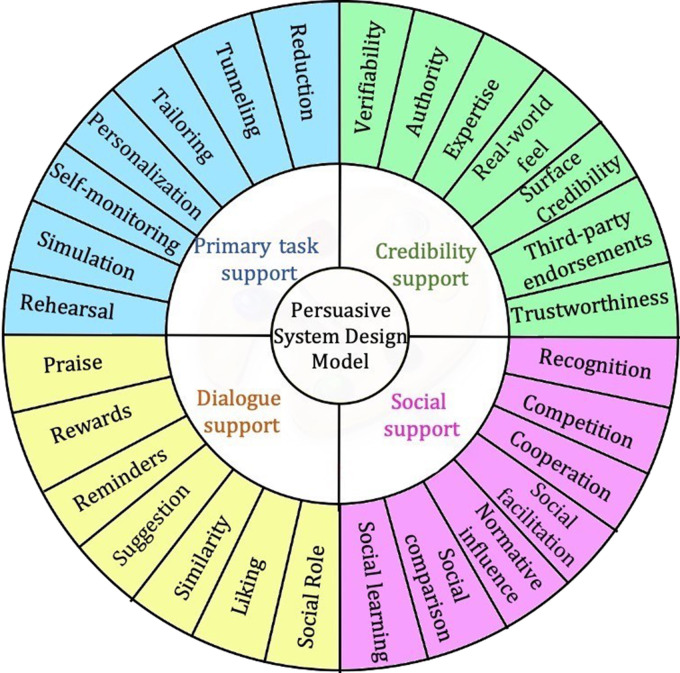
Persuasive system design (PSD) model.^16^

The PSD model suggests the following steps to create a persuasive system, which begin with analyzing the persuasion context. PSD provides 28 design principles or strategies that are categorized into four dimensions of persuasion: (a) primary task support that helps the user carry out their target behavior; (b) dialogue support that uses design principles that motivates the users through feedback and interaction with the application; (c) credibility support uses techniques that make the application look and feel credible to the user; and finally, (d) social support that uses techniques to leverage social influence.^28^Figure 1 illustrates the different techniques of the PSD model. These features can be used to design an engaging and usable e-coaching application to manage caregiving activities. These persuasive design strategies might be effective in influencing people and promoting learning using various persuasive strategies.^29^ They have also been used in e-health to encourage positive change in attitudes or behavior among users.^30,31^ A persuasive e-coaching application uses technology during coaching to encourage and stimulate changes in users’ attitudes and behaviors by leveraging goal-setting and self-management components.^32^ However, the PSD model is a generic model for designing persuasive interventions, but user experiences and interactions differ based on their context.^33^ A recent study indicates that the PSD model needs to be extended based on the use and user context.^34^

This study identifies the unmet needs of caregivers in Sweden to provide design suggestions for an e-coaching application using the PSD model. Some of the elicited needs of caregivers could not be translated to design suggestions using the current list of design principles in the PSD model. Therefore, the PSD model was extended to reflect other kinds of persuasion that can be useful, especially in informal caregiving. Friendsourcing and peer mentoring design principles were added to the PSD model.

The primary contribution of this paper is the adaptation of the PSD model for informal caregiving. This adapted PSD model for caregiving can be used to design and implement digital interventions for caregivers. So far, the PSD model has not been adapted to the context of informal caregiving in the literature. This paper also contributes by applying design principles from the PSD model to present design suggestions for an e-coaching application for caregivers. There have only been a few studies that use persuasive designing in the context of caregivers and even fewer that have used the PSD model, including a recent study that presents a prototype of an m-health app focused on self-management for Hispanic dementia caregivers using the PSD model.^18^

Hence, the research questions addressed in this study are as follows:

- *What are the unmet needs of informal caregivers that can be translated to persuasive design suggestions for an e-coaching application?*
- *What would these persuasive design suggestions be?*
- *What adaptations are needed for the PSD model for informal caregivers?*

## Method

In this study, we adopted a qualitative research approach. Consolidated criteria for reporting qualitative research checklist was followed to report on the method and qualitative findings of this study.^35^ To formulate design suggestions, we began with understanding the needs of caregivers for an e-coaching application. We conducted semi-structured interviews with caregivers to elicit these needs and understand their caregiving context. In this section, we present the information on participants and their recruitment. This is followed by the procedure used for data collection and analysis. Finally, the process used to adapt the PSD model is also presented.

## Interview study

### Participants

The study was conducted in Uppsala, Sweden over a period of 3 months from March 2022. Participants included adult caregivers (>18 years) to family and close friends suffering from long-term chronic illness. Caregivers were contacted through caregiver associations such as “Anhörigas Riksförbund” and the rehabilitation department in any municipality in Sweden. “Anhörigas Riksförbund” is the national caregiver support organization that works to improve the living conditions of caregivers. They have multiple regional units that work at county levels. Many caregivers in Sweden also contact the rehabilitation departments that support them through intense caregiving experiences, support such as help to contact occupational therapists, counselors, and information about caregiving. Information leaflets were distributed in these caregiver associations with links to the study at the university's webpage. Caregivers were given a choice to either use the registration form online to fill in their information or they could contact the research team via phone or email. Some caregivers were contacted through snowballing with the interviewed participants. We received interest from 20 caregivers who were willing to participate in the study. However, only 13 of them could be interviewed. Seven caregivers were unable to participate in the study due to other commitments, despite their initial interest. Caregivers that expressed their interest to participate in the study were geographically distributed in Sweden, from Gothenburg to Stockholm to Skåne. Demographic data for the participants and their care recipients are displayed in Table 1. The research team did not have any relationship with the participants prior to the commencement of the study. We have used the American Medical Association's age classification to present the age of caregivers and care recipients.^36^

**Table 1.** Participant caregivers’ and care recipients’ demographic data.

Gender of caregiver	Age of caregiver	Condition cared for	Gender of care recipient	Age of care recipient	Living status
Male	18–40	Bipolar disorder	Male	65–90	Living separate
Male	65–90	Dementia	Female	65–90	Co-habiting
Female	18–40	Bipolar disorder	Male	18–40	Living separate
Female	18–40	Bipolar disorder	Male	18–40	Co-habiting
Female	65–90	Dementia	Male	65–90	Living separate
Female	18–40	Dementia	Male	65–90	Living separate
Female	18–40	Autism*	Female	0–12	Co-habiting
Female	18–40	Cerebral Palsy	Male	0–12	Co-habiting
Female	18–40	Cerebral Palsy	Male	0–12	Co-habiting
Female	65–90	Cerebral Palsy	Female	0–12	Living separate
Female	18–40	Cerebral Palsy	Female	0–12	Co-habiting
Female	65–90	Dementia	Female	65–90	Living separate
Female	65–90	Dementia	Male	65–90	Co-habiting

*Note*. *The care recipient is able to perform basic activities of daily living independently but is dependent for instrumental activities of daily living.

#### Procedure and measures

A semi-structured interview guide was used with open-ended questions.^37^ SP, PÅ, and ÅC were involved in preparing the interview guide, which was approved by the Swedish Ethical Review Board. Interview questions were based on the informal caregiving context such as caregiving tasks, experience, assistance from family and friends, respite care, and help requested from formal healthcare. We also included questions regarding potential help and support needed for caregivers. All the interviews were conducted in Swedish by a research assistant who was employed at the university. SP had several mock interview sessions with the research assistant. They also had detailed discussions before and after the interviews to ensure that the research assistant had a clear understanding of the participants. The research assistant took field notes during the interviews that were discussed with SP to gain a better understanding of the participants. The interviews were conducted on a one-on-one basis and were audio recorded. The research assistant who conducted the interviews held a Master's in Science in Public Health. She also had prior experience in conducting interviews for research projects. After the interviews were conducted, the research assistant transcribed and translated them into English for analysis. While most informal caregivers preferred to be interviewed over an online video conferencing platform due to its convenience and flexibility, some also preferred to do it over a phone call.^38^ The interviews lasted between 60 and 75 minutes. After conducting 13 interviews and examining the transcripts, SP, PÅ, and ÅC deliberated on data saturation, ultimately concluding that it had been satisfactorily achieved.

#### Interview data analysis

Each participant was given a pseudonymized name like IC.W.13, where “IC” stands for informal caregivers, and “W.13” denotes the 13th women participant contacted and interviewed. Data were analyzed using thematic analysis,^37^ as described in Table 2. Data was stored and analyzed using the qualitative data analysis software MaxQDA. An inductive approach was used to explore the needs of caregivers for the e-coaching application.

**Table 2.** The six steps of thematic data analysis as described by Braun and Clarke.^37^

Step 1	Data was gone through repeatedly by SP to become familiar with it while making notes of ideas. Immediately after the interviews, a quick note was made of the participant's context and an interesting thought associated with it.
Step 2	An initial set of codes was created by SP based on features that were interesting to the aim of the research in a systematic manner.
Step 3	These codes were analyzed by SP and ÅC and were looked for broader themes. The most relevant data was gathered for the potential themes.
Step 4	The broader themes were further reviewed and refined by SP and ÅC to make sure it is important to the research question. We also linked the quotes to the relevant themes.
Step 5	The most important themes were selected and defined to explore the needs of caregivers for an e-coaching application.
Step 6	Finally, the themes are described in the results section including the researchers’ interpretation and illustrating quotes.

### Elicitation of design suggestions and adaptation of the PSD model

Once the caregivers’ needs were identified, they were mapped with the PSD model. The description of the need was compared with the description of the persuasive design principles from the PSD model, and a design principle was chosen based on this match.

In case the description of the needs did not map with any potential design principles from the PSD model, we described the design suggestion in detail and checked if any design principles were similar to it. If not, we suggested a new design principle for the PSD model. Once we described the new design principle, we checked if there was any overlap with an existing design principle from the PSD model. Once no overlap was found, we added it formally as a design principle in the extended model.

## Results

**Figure 2.**
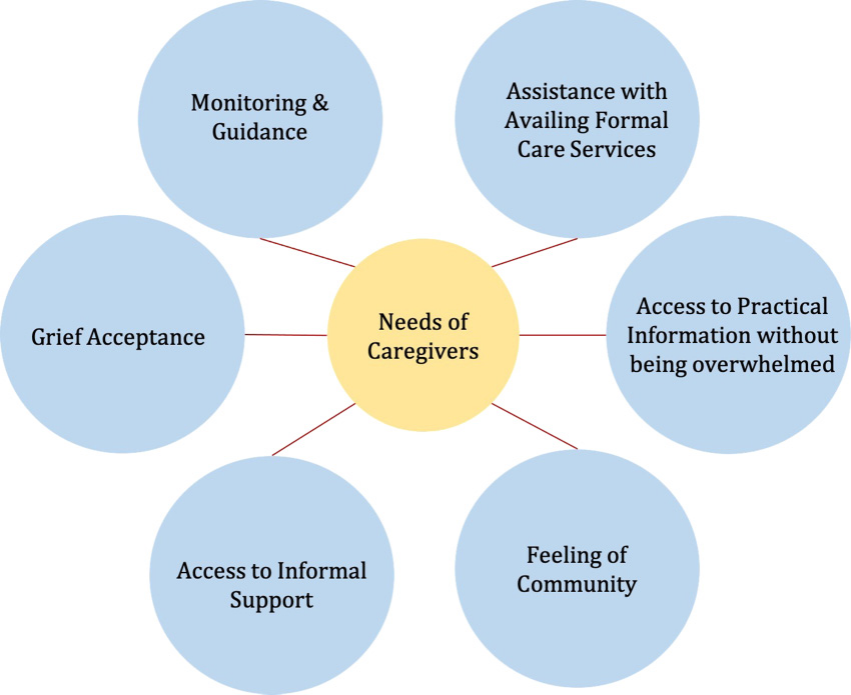
Needs of caregivers identified.

This section describes the caregivers’ needs found from the interviews, followed by the corresponding design suggestions using the PSD model. The adapted persuasive design model for the context of caregivers is also presented. Figure 2 presents the needs of the caregivers identified.

### Monitoring and guidance

#### Description of the need

In our interviews, caregivers expressed a need to monitor the health of their care recipient to make sure there are no major deviations from their health status. They explained that monitoring the care recipient's health status can be easy if they had information regarding checkpoints to track so that the condition of their care recipient is stable over a duration of time. They felt that it would be useful to have some checkpoints or flags about the health of their care recipients that when they encounter them could indicate that corrective measures such as medicines are needed to keep the care recipient healthy. This need was more emphasized by caregivers of patients with mental health illnesses. The caregivers emphasized the significance of this need, as they recognised that each time a care recipient is hospitalised, it generates negative experiences for both the caregiver and the care recipient, in addition to incurring costs for formal healthcare.

No, but it's like this, at some point they [formal healthcare] have said that they will work to flag concerning behaviour earlier, so when these flags come up then we’ll do this, this, and this, so it becomes like a, a yes how do you put it, a tactic eh to try to catch it in time. But to achieve that then it must, and I have said this several times, the healthcare system must be responsive. **(Male, father with bipolar disorder, 47)**

#### Design suggestion using persuasive design

To address this need, we suggest the reformulated “monitoring” design principle be used along with the “suggestion” principle of PSD. The monitoring design principle is different from the “self-monitoring” design principle of the PSD model, where the user makes use of certain checks to monitor their own progress. In the “monitoring” design principle, the caregivers can monitor the health status of their care recipient. The caregivers can enter important checkpoints and monitor them. As and when caregivers encounter them, they can check them off, and the application can suggest steps needed in such situations. For this function to work, a strong collaboration with formal healthcare in terms of keeping the caregivers updated on the red flags of illnesses and measures to correct them is needed.

### Assistance with availing formal care services

#### Description of the need

Most caregivers emphasized the importance of formal services that support them in performing their caregiving tasks. They stressed on the complexity of the formal healthcare system. However, they pointed out that there is much confusion and ambiguity in their understanding of what kind of services they are entitled to and the processes to be followed to avail of them. They also discussed their sheer frustration in not knowing when to avail of these formal services and indicated their need to have some assistance in this regard. They also seemed to need help maneuvering the bureaucracy of the system.

(One) should get the help needed and someone needs to inform you on what the next step is, what you are entitled to. There's no one to help you with it, so all the time you have to look for that information by yourself and it's also very time consuming, and takes a lot of energy. /…/ Nothing was coordinated, there isn’t someone for patients who have a lot of care needs, and there isn’t someone who has overall responsibility. **(Female, 34, daughter with cerebral palsy)**

#### Design suggestion using persuasive design

The “reduction” design principle of PSD can be used to address this need. This design principle reduces the effort spent to complete a task. Here, it can be used to make it simpler for caregivers to know the formal services available for them at a given stage and how to access them. The e-coaching application can provide caregivers with suggestions of formal services to which they are entitled. Once they select a particular service, a step-wise tutorial on how to apply for the service can be provided along with information on supporting documents required. The e-coaching application can also provide important points to remember, for instance, how to proceed if the application for personal care assistance is rejected. Therefore, the effort that caregivers invest in identifying services they are entitled to and knowing the process is reduced.

### Access to practical information without being overwhelmed

#### Description of the need

Most caregivers go through different stages of caregiving during which their information needs change, and they may need different kinds of formal services to assist them in their caregiving activities. They stated that this was mainly due to changes in the condition of their care recipient. In our interviews, too, we found that sometimes caregivers found themselves in situations where there was a lot of information at different levels (basic to advanced). Most felt they needed to invest more time to find relevant and timely information. For instance, on Facebook groups, caregivers may have to scour through vast amounts of posts and information to find something relevant.

I have read a lot myself, /…/ received a lot of information from dementia teams from [relative consultants]. Because it's very heavy and sad to see these changes happening gradually. I still work full time so it has become a lot for me. I have visited my mother several times a week on the weekends and [she] constantly goes into different stages this with dementia, so information I need changes all the time. **(Female, 64, mother with dementia)**

#### Design suggestion using persuasive design

Most caregivers would benefit from access to practical information about caregiving without being overwhelmed. To ensure the presentation of relevant and timely information to caregivers, the e-coaching application can provide information in various stages. This can be addressed using the “tunneling” design principle of PSD. “Tunneling” is usually used to hide the complexity of the application in order not to overwhelm the user.^28^ In this case, caregiving information is provided in stages to caregivers, from simple to more advanced. When caregivers need access to some information, simple information for that particular caregiving level is provided first. Access to advanced information is displayed only after caregivers choose to view it. This way, caregivers can control the level of information they view and avoid information overload.

### Feeling of community

#### Description of the need

Most caregivers reported that they spent long hours providing care, and many did not have a functioning social group. A social group is known to provide people with a sense of belonging and is also a platform for sharing information. Most caregivers emphasized that being a part of a social group has helped them get a lot of information from other caregivers that they did not receive from the healthcare system. They also felt that they couldn’t reflect on their feelings while engaged in caregiving but talking to other caregivers allowed them to process their feelings. Most caregivers interviewed mentioned that they would like to have a place where they can discuss their caregiving situation and ask questions or just have a place to vent out their frustration. They believed that this kind of support was important to also know what to expect in the caregiving process.

(have) contact with other parents that's where you get all the information that you don’t get through the healthcare system. /…/ You definitely need someone to vent with because there is so much that you push away at home because you don’t have time to reflect on your own well-being or to even feel. **(Female, 34, daughter with cerebral palsy)**

#### Design suggestion using persuasive design

The persuasive design principle of “social learning” is used to address this. A forum platform can be provided with separate sections to share their experiences, ask questions, and vent their frustration, anonymously if needed. They can also form smaller groups based on their geographic location or care recipients’ medical condition, among others. Connecting and chatting with individual caregivers over the application is another possible feature. This way, they can feel part of a community and learn from each other and also feel in control of their physical and mental health.

### Access to informal support

#### Description of the need

In our interviews, most caregivers expressed the need to have access to volunteers or informal support in their neighborhood or among their informal social network for extra informal assistance. Some caregivers added that during the pandemic, such networks in their neighborhoods worked well. They also reported that caregiving can be a lonely journey that can sometimes be very consuming of time, effort, and emotional health. While many of their friends aimed to be considerate of their situation by refraining from diturbing them, caregivers expressed their preference for close family and friends to periodically inquire about how they can be of assistance, rather than simply leaving them alone.

The help and support can be that you can take out (his) elder sister to go and get ice cream. People don’t understand that! And it's precisely that form of support that would be such a relief for us! (With) everyday things, like going to recycling, cleaning up. You can help in a million different ways. But no, you can’t help us with a cough machine and if our child gets a mucus plug. **(Female, 31, daughter with cerebral palsy)**

#### Design suggestion using persuasive design

This need can be addressed by having a provision for care volunteers or family and friends of caregivers to register themselves in the e-coaching application as care volunteers. “Friendsourcing” is a design principle added to the PSD model that encourages or motivates people in caregivers’ neighborhoods to provide informal support to caregivers. Caregivers can upload a list of tasks on their account and volunteers can choose which task they can provide assistance with. This brings together caregivers and care volunteers on a single platform to collaborate on tasks. The application can also suggest tasks to care volunteers or family and friends based on the parameters such as the last task they helped with, or the last caregiver they assisted, etc.

### Grief acceptance

#### Description of the need

Most caregivers expressed that they find it difficult to come to terms with their situation. Some felt anger and a sense of unfairness at the health situation of their care recipients, especially when their care recipients were children. Many stated that they have been experiencing a lingering sense of grief for a long time and added that suffering from grief about this situation can be very engulfing. For many, this had a negative effect on other relationships such as with a spouse or with their parents. Most of them are looking for help in accepting grief or the situation that they are dealing with.

I think everyone has been very empathetic and supportive. And are super sweet and have helped with fundraisers. But at the same time, it's also difficult when everyone is sitting there with their healthy grandchildren and like… There is a future for them. You have like a grief all the time. I meet those who have gone in CBT-based [therapy] so that is not the solution either. I believe in a fellow human being who listens and can stand to hear that grief and frustration. **(Female, 62, granddaughter with cerebral palsy)**

#### Design suggestion using persuasive design

This need is addressed by a “peer mentoring” design principle, which has been added to the PSD model. In “peer mentoring,” users can be motivated to do a targeted behavior by interacting with someone (like a mentor) who has been in a similar situation on an individual basis. These mentors could support them, and provide them with information and material that they have found useful. When a caregiver registers themself in the application, they are asked if they want to be a mentor to another caregiver. Caregivers can view all other mentor caregivers and choose to contact those whom they feel comfortable talking with. The application can also provide suggestions such as matches on mentors based on the caregiving condition, relationship with the relative, and other relevant parameters.

### The adapted persuasive design model for informal caregivers

**Figure 3.**
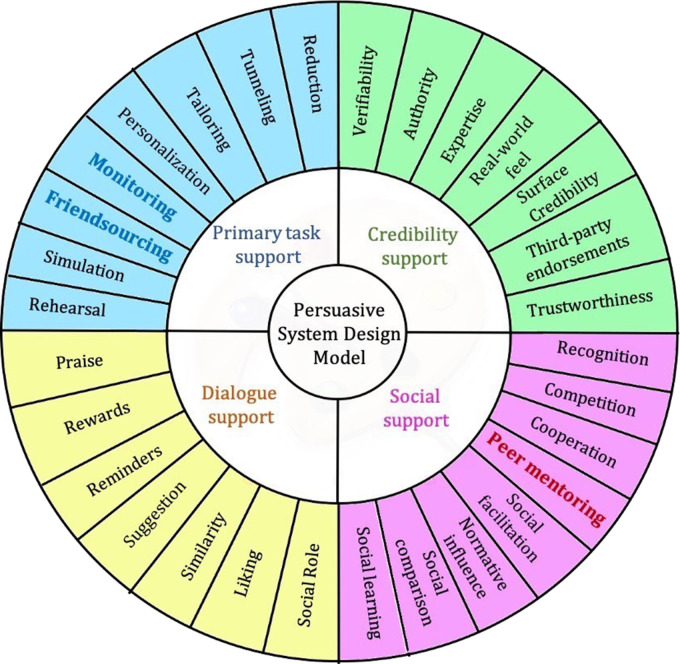
The adapted persuasive design model for informal caregivers.

Some of the needs such as *monitoring and guidance*, *access to informal support,* and *grief acceptance* could not be mapped to any specific existing design principles in the PSD model. Hence, we extended the PSD model for informal caregivers as illustrated in Figure 3. For the need *monitoring and guidance*, the closest design principle in the PSD model is “self-monitoring.” In self-monitoring, users can monitor and track their own performance or status. In the context of caregiving, there is a need to be able to also track and monitor their care recipients’ health status. For this reason, we reformulated the “self-monitoring” design principle to “monitoring” to reflect this change in focus.

For the need *access to informal support*, caregivers wanted to be able to receive assistance from their family and friends or volunteers with some tasks. This design principle of being able to persuade a user by providing informal assistance in the form of sharing some tasks is not addressed in the existing PSD model. Hence, we introduced a design principle called “friendsourcing.” Here, users can find informal assistance for chores or tasks that they need help with. They can also share these chores or tasks with their family and friends or care volunteers.

In addition, for the *grief acceptance* need, caregivers wanted to be able to connect with other caregivers who have been in their situation. They wanted to be able to ask them questions, perhaps have some information that worked for other caregivers, or just be able to share their grief with someone who has gone or is going through something similar. This one-on-one interaction/mentorship is not addressed in the existing PSD model; hence, we introduce the design principle “peer mentorship.” The design principles from the PSD model that were similar to “peer mentorship” are “social facilitation” and “normative influence.” However, “social facilitation” does not include the focused attention of “peer mentorship,” and “normative influence” focuses on influence and pressure rather than mentorship and just being there.

## Discussion

In this study, the needs of informal caregivers were explored, based on which design suggestions for an e-coaching application were presented. Through our qualitative findings, six needs emerged: monitoring and guidance, assistance with availing formal services, access to practical information without being overwhelmed, feeling of a community, access to informal support, and grief acceptance. The PSD model was then used to outline design suggestions for these caregivers’ needs. The PSD model is a generic model to design interventions; however, user interactions and experiences are mainly contextual, which can differ depending on circumstances and dynamics.^33^ Furthermore, this study also found that the current PSD model needed to be extended and reformulated to fit the context of informal caregiving. Hence, we adapted the PSD model for informal caregiving by adding new design principles to it. Designers and information system researchers can use this extended PSD model to design IT-based applications for caregivers. Interestingly enough, the design suggestions based on the PSD model are mostly from the primary task support and social support groups indicating that the main needs of caregivers are pertaining to the caregivers' primary task and social aspects. Since this a generic e-coaching application, most of the needs identified pertain to general needs of caregivers related to access to information, formal services, informal support, and connected community. More focused studies are needed to elicit specific needs of caregivers who provide care to children or older adults. The resulting e-coaching application would then be more specific to the condition that the caregiver provides care for.

The findings of this research suggest that persuasive designing can be used to present design suggestions for an e-coaching application based on caregivers’ needs. The PSD model has also been adapted to suggest design suggestions for the caregiving context. Below, the findings are discussed in the context of the PSD model and other theoretical literature, pointing to some limitations of the study.

Caregivers would like to monitor the health status of their relatives. Hence, here the “self-monitoring” design principle was reformulated to the “monitoring” design principle. This updated design principle can be used while developing applications that need to monitor the status of someone other than the user. We suggest having this principle in the PSD model instead, as it can address a wider range of application needs. However, the literature suggests that physicians have been reluctant to share medical information with patients and their caregivers due to the anxiety it may cause.^39^ Furthermore, on many occasions, patients are hesitant to share their illness information with family members to avoid causing them worry.^40^ Therefore, “monitoring” needs to be used with discretion while also addressing the question of privacy.^41^

Recent literature has shown that caregivers need timely and easy access to formal care services to manage their caregiving burden.^42,43^ Our study proposes to provide a step-wise guide to availing formal services for caregivers depending on their caregiving stage and current need. However, to be able to provide a step-by-step guide to availing these services, caregivers need to be aware of these services. Various formal services may be scattered in different sectors of the social security system,^44^ which is very complex and confusing to caregivers.^43^ Hence, these processes to avail of services need to be optimized. Given the stress of caregiving that the caregivers already deal with, such processes need to be easily understood. However, the dwindling availability of these formal services and changing eligibility criteria are often stated as reasons for caregivers’ inability to use these services. Past studies also indicated that this inflexibility of formal services has been a major concern for caregivers,^43^ adding to their stress, frustration, and helplessness. Research shows that many informal caregivers need much more hours of assistance from formal caregivers but the formal care system is not equipped to handle this due to decreased investments in healthcare.^45^

Most caregivers in our study suggested a need to access relevant information without being overwhelmed. However, this information varies throughout the caregiving journey.^46,47^ Many caregivers also feel lost in the information that is available on the Internet, which can be challenging. Hence, we recommended the use of the “tunneling” design principle to present relevant information proactively in multiple stages. This is one of the most important needs of caregivers. However, the literature consistently reports a lack of adequate practical information available to caregivers.^48^ Also, there is evidence that most caregivers need efficient delivery of this information from trustworthy sources.^49^ Caregivers also preferred that this information be easy to access.^49^ Providing practical and trustworthy information through “tunneling” to caregivers addresses these concerns. This may also reduce the effort that users put into using the application.^50^

Our findings show that caregivers may feel socially isolated due to their caregiving responsibilities and would benefit from being part of a community. Feeling part of a community can help reduce the social isolation many caregivers may suffer from.^51^ Studies also showed that most caregivers tended to turn to online applications to find these communities and felt a sense of social belonging through them.^52^ Previous literature on persuasive designing indicated that “social comparison” and “competition” design principles are preferred in e-health applications.^53^ However, since caregivers are vulnerable users, using these design principles might cause them further stress. Instead, this study proposes “social learning” as a way for caregivers to collectively learn about each other's caregiving experiences and progress in their caregiving activities.

Most caregivers indicated that they need informal help from friends and family regarding informal, non-caregiving tasks such as grocery shopping, picking up children from school, etc. A recent study suggests that among the five dimensions of social support, namely, informational, esteem, emotional, tangible, and belonging, caregivers highly requested tangible support.^54^ Tangible support (or in this study, informal support) is the help provided to caregivers in terms of goods or services such as buying a wheelchair or helping with the grocery.^54^ Past literature suggests that such support from friends and family or volunteers from their neighborhood is the best source to relieve some stress.^55,56^ Informal support can also improve the quality of care that caregivers are able to provide to their relatives, as they may be more able to focus on the needs of the care recipient, rather than being overwhelmed by the demands of caregiving. This can ultimately lead to strengthening of the caregivers’ well-being.^56,57^ To this effect, we propose to add the “friendsourcing” design principle to the PSD model. “Friendsourcing” has been found to be effective in caregivers by providing support and reducing stress.^58^ Applications such as CaringBridge based on “friendsourcing” design principle that leverage social media to provide informal support have been hugely successful among caregivers.^59^

Our findings also indicate that most caregivers experience grief in their caregiving journey and find it difficult to accept the situation. Some interviewed caregivers struggled with accepting their children's disability at birth and felt that it was unfair that their children may live out “lesser lives.” Past research also acknowledges that most caregivers may find it difficult to deal with the uncertainty and being a sudden caregiver.^45,60^ Some felt an ambiguous loss that their relative is not mentally there anymore (in dementia and Alzheimer's caregivers), which negatively affected their well-being.^61^ Negative caregiver well-being may lead to frequent hospitalizations increasing the cost to formal healthcare.^61,62^ Therefore, they need support in accepting such situations and coping with grief. Such support has been found in the literature to be important for the caregivers’ well-being.^63^ “Peer mentoring” is added to the PSD model to provide users with focused and individual mentorship with other users in the same situation.

### Limitations and future research

Elicitation of user needs is an iterative process, and we present results from only the first iteration here. This study is part of a larger research project, and further studies will focus on eliciting and expanding on the additional needs of caregivers. In our study, we did not find any difference in the needs of caregivers based on the age of the care recipient or the condition they cared for. However, we provide design suggestions for a generic e-coaching application, which may limit the specificity of information and coaching that can be provided compared with an e-coaching application focused on a specific condition. Hence, future research can focus on designing e-coaching applications for specific care-recipient conditions or specific kinds of caregivers, such as young adult caregivers or young care recipients. There are other conditions where caregivers provide care such as stroke and cancer, but we could not include them in our study owing to the inaccessibility of these caregivers. Future research could be more representative in this regard. We have introduced an extended PSD model for informal caregiving. A limitation of this work is that this extension is based on interviews with a small group of caregivers. More empirical evidence is needed to explore the implications of this extended model in detail. Also, the extended PSD model for informal caregiving is not tested for usability and user experience (UX); therefore, we cannot validate the extended PSD model. Future research can focus on validating the extended PSD model in the informal caregiving context.

## Conclusions

In this article, we uncovered caregivers’ needs and proposed design suggestions based on the PSD model. We found that the design principles in the PSD model may not be sufficient to propose design suggestions adapted to the caregiving context. Hence, we reformulated the “self-monitoring” principle to the “monitoring” design principle. We also added two design principles, namely, “friendsourcing” and “peer mentoring” to the PSD model. Our extended model and the design suggestions are expected to provide some important guidelines to researchers and designers for developing e-health applications with a clear focus on users and their needs. This adapted PSD model for the caregiving context can be used to design persuasive digital interventions for caregivers.

## Supplemental Material

sj-docx-1-dhj-10.1177_20552076231177129 - Supplemental material for Design suggestions for a persuasive e-coaching application: A study on informal caregivers’ needs Click here for additional data file. Supplemental material, sj-docx-1-dhj-10.1177_20552076231177129 for Design suggestions for a persuasive e-coaching application: A study on informal caregivers’ needs by Shweta Premanandan, Awais Ahmad, Åsa Cajander, Pär Ågerfalk and Lisette van Gemert-Pijnen in DIGITAL HEALTH
