# Supplementary material for: Design suggestions for a persuasive e-coaching application: A study on informal caregivers’ needs
Source: Digit Health. 2023 May 30;9:20552076231177129. doi: 10.1177/20552076231177129 (PMC10240856; doi:10.1177/20552076231177129)
Supplement: sj-docx-1-dhj-10.1177_20552076231177129 - Supplemental material for Design suggestions for a persuasive e-coaching application: A study on informal caregivers’ needs [file sj-docx-1-dhj-10.1177_20552076231177129.docx]

**Semi-structured interview questions - Informal caregivers**

- Can you describe a normal day?

Follow-up questions:

- - How long do you provide care? Intensity
  - Relation to the care recipient? Cohabitation care or distance care?
  - How long have you been providing care?
  - In what way do you feel that you are then supporting your relative.
- How do you think that caregiving affects your daily life?

Follow-up questions:

- - Are you able to take time for yourself? (Give examples)
    - Examples if yes
    - If no, what kind of self-care activities would help you?
- What type of help are you seeking or would you like to receive from caregiver organizations? (Such as these Anhörigas Riksförbund, Anhörigstöd in the Regions)

Follow-up questions:

- - How did you become aware of any services?
  - Has it helped you?
  - If not, what kind of help would you need?
  - Have you sought help in this regard from anyone? Any support organizations?
- Are you able to seek support from your family and friends?

Follow-up questions:

- - What type of support and for how long?
  - Why not?
- Have you taken a break from caregiving?

Follow-up questions:

- - How do you feel about it? For how long? How did you feel after that?
  - Have you felt compelled to take a break due to the caregiving situation?
  - Why not?
- How is your experience about using any IT or computer-based support for caregivers?

Follow-up questions:

- - What do you think about these support systems? Who provided them?
  - Do you need any other type of help?
  - If no, why not?
  - Do you feel comfortable using computer-based support?
  - Does it worry you?
  - What other functions would you like to see?
- What are your needs for support in the caregiving role and also for your own needs? What any other kind of support do you need?
